# Dr. William J. Tuck

**Published:** 1859-09

**Authors:** 


DR. WILLIAM J. TUCK, a native of Virginia, died at Memphis, of gastro-enteritis, on the 8th of June, in the forty-fifth year of his age. He was a graduate of the University of Pennsylvania; and during the past session held the position of Professor of the Institutes of Medicine in the Memphis Medical College.

